# Sleep Modelling across Physiological Levels

**DOI:** 10.3390/clockssleep1010015

**Published:** 2019-03-04

**Authors:** Svetlana Postnova

**Affiliations:** 1 School of Physics, University of Sydney, Sydney 2006, NSW, Australia; svetlana.postnova@sydney.edu.au; 2 Center of Excellence for Integrative Brain Function, University of Sydney, Sydney 2006, NSW, Australia; 3 Charles Perkins Center, University of Sydney, Sydney 2006, NSW, Australia

**Keywords:** sleep, circadian clocks, mathematical modelling, behaviour, mean field, neurons, molecular mechanisms, EEG, systems biology, multi-scale

## Abstract

Sleep and circadian rhythms are regulated across multiple functional, spatial and temporal levels: from genes to networks of coupled neurons and glial cells, to large scale brain dynamics and behaviour. The dynamics at each of these levels are complex and the interaction between the levels is even more so, so research have mostly focused on interactions within the levels to understand the underlying mechanisms—the so-called reductionist approach. Mathematical models were developed to test theories of sleep regulation and guide new experiments at each of these levels and have become an integral part of the field. The advantage of modelling, however, is that it allows us to simulate and test the dynamics of complex biological systems and thus provides a tool to investigate the connections between the different levels and study the system as a whole. In this paper I review key models of sleep developed at different physiological levels and discuss the potential for an integrated systems biology approach for sleep regulation across these levels. I also highlight the necessity of building mechanistic connections between models of sleep and circadian rhythms across these levels.

## 1. Introduction

**Figure 1:**
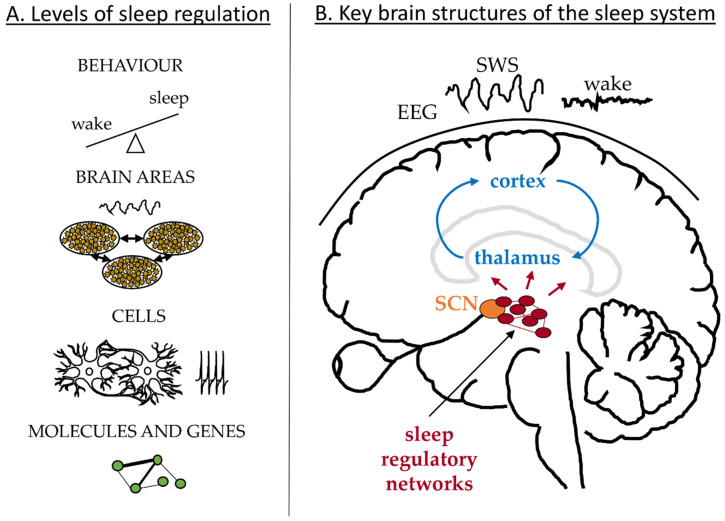
Schematic of the key systems involved in regulation of sleep. **A**: Levels of sleep regulation from molecules and genes to cells, brain areas and behaviour. **B**: Key brain structures addressed in this review: cortex, thalamus and the areas of the sleep regulatory network of the hypothalamus and brainstem.

Sleep is manifested and regulated across multiple physiological levels [1] as illustrated in Figure 1A. On the behavioural level our states of wakefulness and sleep change with a period of 24 h and can be affected by external or internal factors leading to sleep debt and misalignment between sleep and circadian clocks; for example due to shift work or jetlag. These are associated with changes in the brain dynamics recorded in the EEG. Sleep deprivation results in an increased SWA during recovery sleep and increased power in the theta band during extended wakefulness [2,3,4]. The EEG dynamics depend on the electrical activity of neuronal networks in the cortex and thalamus as schematized in Figure 1B, which change their firing pattern and synchronization state along the states of sleep and wakefulness [5,6,7,8]. Deeper in the brain, neurons in the hypothalamus and brainstem control the switch between the sleep and wake states and send projections to the thalamus and other brain areas [9,10], Figure 1B. The switch itself is regulated by the circadian and homeostatic processes in line with the two-process model of sleep regulation [11,12]. The aim of this paper is to review mathematical models addressing sleep phenomena across these different physiological levels and across different brain structures. In the following I briefly review relevant physiology of sleep regulation and then discuss existing models according to the physiological levels they focus on. 

The circadian system controls the 24 h periodicity of physiological functions, including sleep [13,14,15]. The master circadian clock is located in the SCN of the hypothalamus and is entrained to exactly 24-h period by the light-dark cycle [16]. It is most sensitive to the short wavelengths of light (peak at 480 nm) and the effects of light on the SCN are realized via activation of the melanopsin-containing ipRGCs in the eye, see for example, [17,18]. In addition to the master clock in the brain, peripheral circadian clocks are found in other organs, including liver, pancreas and muscles and are proposed to be synchronized by the SCN and entrained by non-photic zeitgebers such as food [19,20]. At the genetic level, the circadian rhythm arises from the nearly 24-h cycle of the genes’ translation and transcription feedback loops. The key molecular components of these genetic networks and their interaction were identified and described in great detail, see [13,21,22] for reviews. Each SCN neuron has this genetic machinery inside, which leads to daily fluctuations in spiking activity even in isolated SCN neurons [23,24]. Coupling between the SCN neurons in the brain allows them to synchronize to the same period with high firing activity during daytime (light) and low during the night (dark) [25]. SCN sends synaptic connections to other brain and peripheral areas to ensure alignment of different rhythms [19]. In diurnal animals, including humans, high activity of the SCN is wake-promoting and a consolidated sleep is associated with low activity of the SCN [13]. 

The homeostatic process regulates sleep debt with sleep pressure increasing during wakefulness and decreasing during sleep [11,12]. If wake is extended, the homeostatic process leads to a rebound of sleep duration at the behavioural level. At the level of mean field EEG, increased sleep debt is reflected in an increased intensity of SWA, which serves as the main marker of sleep homeostasis [2,11]. At the cellular level, SWA arises from the dynamics of neuronal networks in the cortex and thalamus, which change their firing pattern along the states of sleep regulatory networks [5,6,7,8]. Synaptic potentiation in cortical networks during wakefulness and pruning during sleep are proposed to be at the core of the homeostatic regulation of sleep [26,27] but several other mechanism may also be involved. For example, activity of the neurons in the cortex, thalamus and the sleep-regulatory networks, is modulated by a variety of chemicals, some of which are found to affect SWA and proposed to play a role in sleep homeostasis. These include adenosine, nitric oxide, prostaglandine D2, tumour-necrosis factor, interleukin-1 and growth-hormone-releasing hormone [28]. Finally, homeostatic regulation is under genetic control [29] and several molecular level biomarkers whose expression depends on time awake were identified [30,31,32]. All these findings across different physiological levels are yet to be reconciled into an integrated theory explaining sleep homeostasis. 

Together, the circadian and homeostatic processes control the timing and structure of sleep, with the longest consolidated sleep duration observed during the biological night (low circadian activity in humans) [11,33]. These two processes were first expected to be independent of each other, because lesion of the SCN in rats did not affect SWA, even though it destroyed the periodicity of the sleep-wake cycle [34]. However, an increasing number of studies show evidence of interaction between the two processes at different physiological levels [35,36,37]. For example, at the behavioural level, sleep deprivation attenuates phase shifting of the circadian clock due to light [38,39]. At the EEG (mean field) level, the SWA is modulated by the circadian phase, in particular in the frontal brain areas [40]. Sleep deprivation and thus increased SWA during rebound sleep, lead to a decrease in neuronal activity of the SCN during NREMS [41]. At the molecular level, mice lacking core clock genes (e.g., BMAL1, CLOCK, CRY) show disturbances in sleep homeostasis, while sleep history affects expression of clock genes in the mouse forebrain, see [42] for review. 

Overall, a number of processes across different physiological, temporal and spatial scales are involved in regulation of sleep. These are likely to interact with each other both at the same physiological level and across the levels. The connections between these levels, however, are poorly understood, which is partly due to methodological differences of experimental research at different scales. Mathematical modelling provides us with a tool to integrate the variety of seemingly disparate experimental data across the physiological levels to make testable predictions and develop theories. 

Modelling plays an important role in the fields of sleep and circadian research with benefits ranging from advancing our fundamental understanding of the mechanisms to guiding experiments and making predictions about sleep, circadian and alertness dynamics [36,43,44,45,46,47,48,49,50,51,52,53,54,55,56,57,58]. However, similar to experiments, models of sleep and circadian dynamics so far mostly focused on a specific physiological level including behavioural, mean field and cellular activity. 

Interestingly, the studies of circadian rhythms and sleep regulation have mostly proceeded in parallel, with only occasional overlaps. Models of the circadian clocks do not usually incorporate sleep, if it is considered then only as an output; while models of sleep regulation usually use a simplified representation of the clock (e.g., a sine function or an abstract oscillator) to simulate sleep timing or do not consider the clock at all. In the past five decades more than 600 theoretical studies were published in each of these fields. A number of detailed reviews of circadian models have been published [44,45,46,47,48,49,50,55,56,57,58,59], including those focusing on modelling at different physiological levels and highlighting the need for a systems theory approach for understanding the circadian system [49,55,56]. Models of sleep regulation have been reviewed separately. These reviews focused mainly on the behavioural sleep-wake patterns and the two-process model [36,43,51,52], neural mass models of sleep regulatory networks [53] and a thermoregulatory model of sleep [54]. Thus, a comprehensive review of the models across the different physiological levels is lacking. 

In this review I focus on modelling of sleep regulation across different physiological levels, including behavioural, mean field level of interacting brain areas and cellular level. Due to the large number of modelling studies I narrow down my overview to models of mechanisms responsible for sleep-related physiology but do not include applications of these models; for example, to shift work, jetlag or prediction of alertness – these deserve their own review [60,61,62,63]. I give examples of the models at each of the physiological levels and discuss differences in their use and methodology. 

## 2. Behavioural Level 

Models at the behavioral level aim to uncover the key concepts undelying sleep-wake behavior and explain sleep patterns observed under a variety of conditions, including normal sleep, sleep deprivation, sleep at different circadian times, free-running sleep under constant light conditions and spontaneous internal desynchrony. The key output of these models is the timing of sleep compared to circadian rhythm. Model variables in this case are not typically assigned to specific physiological measures but can be qualitatively related to underlying brain dynamics. Examples are accumulation of unspecified sleep-promoting substances for the homeostatic process and core body temperature rhythm or electrical activity of the SCN for the circadian drive. Some of the key models at the behavioral level are summarized in Table S1.

The seminal two-process concept of sleep was proposed in 1982 by Alexander Borbély [11]. It was then formalized into a mathematical model [12,64] and has shaped how we view sleep regulation today. This model introduces the homeostatic sleep process based on the intensity and rate of change of NREMS SWA in sleep EEG. It postulates that sleep timing is determined by the interaction between the homeostatic (S) and the circadian (C) processes. Process S is modelled as an exponential decline of sleep pressure during sleep and its increase during wake with the time constants derived from the rates of change of SWA during regular sleep and during recovery from sleep deprivation in humans. The physiological origin of the process S could be an accumulation of sleep-promoting substabces in the brain and their clearence during sleep but these are yet to be identified. Process C represents the dynamics of the master clock in the SCN and is modelled as two skewed sinusoid threshold functions. Sleep onset is triggered when S reaches the upper threshold of C and wake when S reaches the lower threshold [12]. 

Despite its simplicity the model explains a large number of sleep phenomena, including rebound in duration of sleep and increase in SWA after sleep deprivation, circadian modulation of sleep propensity, sleep fragmentation and spontaneous internal desynchrony. The two-process model was later extended to include ultradian dynamics for NREMS-REMS cycles during sleep [65], refined to enable quantitative prediction of SWA [66] and adjusted to account for rodent sleep [67]. Modifications were made to introduce circadian variation of the homeostatic process [68]—the existence of which was later supported by forced desynchrony studies [40]. For detailed reviews of the two-process model see [36,43,51]. 

The two-process model is widely used to explain sleep dynamics, design new experiments and make predictions. It is an integral part of the more recent neurobiology-based models of sleep regulatory networks (see Section 3.2) and is the basis for beavioral level models of sleep propensity, alertness and performance reviewed elsewhere, see for example, [62,63,69,70,71,72,73,74,75]. Most of these models assume additive effects of S and C on sleep-related phenomena, while some proposed nonlinear interaction [73,76]. The exact nature of this interaction, however, is yet to be fully determined [36].

Around the same time that the two-process model was proposed, several models employing multiple oscillators were published and explained many of the key circadian features of sleep, including dynamics under constant light conditions (free-running), sleep duration depending on the circadian phase and spontaneous internal desynchrony between sleep and temperature rhythms [77,78,79]. These focused on the circadian rhythm of sleep as opposed to SWA dynamics in the two-process model and their further development and modifications focused primarily on the circadian dynamics; for example, phase response and dose response to light [80,81].

Two more recent models focused on thermoregulatory and energy-saving hypotheses for the function of sleep. The thermoregulatory model of NREMS control [54,82] proposed that the main function of sleep is to reduce the heat load in the brain which increases due to wakefulness and was able to reproduce key experimental data for sleep deprivation, internal desynchrony and polyphasic sleep. Another hypothesis for the function of sleep is that sleep saves energy by reducing metabolic rate. This idea was addressed in a recent model aimed to resolve the discrepancy between reduction of metabolic rate and the experimentally-observed upregulation of a variety of functions during sleep [83]. It proposed that the state-dependent partitioning of metabolic processes rather than direct reduction of metabolic rate is the primary mechanism for reduced energy use in sleep.

Overall, behavioral level models of sleep are instrumental in establishing a way of thinking about the system and identifying the key components and interactions controlling the sleep-wake cycles. Once such a framework is established, understanding the complex system of numerous interacting components across the physiological levels of sleep becomes more attainable because the different lower-level components can be viewed as contributng to or being affected by, the higher level functions; for example, the circadian or homeostatic processes. It is likely that more than one concept at the behavioral level is required to account for the different aspects of sleep dynamics and functions; for example, accumulation and decay of sleep debt versus state-dependent energy use or temperature change, so the existing models are not necesserily mutually exclusive but can be complementary to each other.

## 3. Brain Areas—Mean Field Dynamics

Models at the level of interacting brain areas aim to explain the role of neuronal populations and their connections in generation of sleep-wake patterns and large-scale brain dynamics such as EEG. Neural field and neural mass modelling approaches are often used at this level to simulate dynamics of neuronal populations, including their mean firing rate and mean voltages. These approaches do not account for the dynamics of individual neurons, instead focusing on the dynamics of a population as a whole. The neural field method allows for travelling waves in the spatial domain, which is relevant for modelling cortical activity, while neural mass treats each population as being too small for waves’ propagation. The key outputs of these models are the mean voltages, firing rates and the EEG spectra and time series, so they can be directly compared to experimental EEG data. The models at this level can be further divided into those focusing on large-scale brain dynamics in the cortex and thalamus (Section 3.1) and the sleep regulatory networks (Section 3.2).

### 3.1. Large-Scale Brain Dynamics—Cortex and Thalamus

Large-scale mean field models of sleep mainly focus on the mechanisms responsible for generating sleep features observed in the EEG. These include the SWA during deep NREMS, spindles and K-complexes observed in sleep stage 2, transitions between different sleep stages including NREMS and REMS and transitions between sleep and wake. Since the EEG reflects the dynamics of cortical neurons, the large-scale models mainly focus on dynamics of the cortex and the interaction between the cortex and thalamus – the so-called thalamocortical, also called corticothalamic, loop (the choice of the term depends on which brain area authors consider to be driving the dynamics). Notably, majority of such models are developed to study a variety of mean field brain phenomena arising from the dynamics of cortex and thalamus, with sleep being only one of them. Some of the key models at this level and their features are summarized in Table S2.

Majority of these models simulate the interaction among excitatory and inhibitory cortical populations and thalamic relay and reticular nuclei involved in generation of the EEG [84,85,86,87,88,89,90,91,92,93,94,95]; some of the models account for cortical dynamics without explicitly incorporating the thalamus [96,97]. Collectively, these models reproduce sleep and wake EEG features and propose mechanisms for how these features are generated at the level of interacting neuronal populations. For example, the corticothalamic model by Robinson et al., proposed that corticothalamic and intrathalamic loop resonances account for the alpha, beta and spindle peaks and for the changes in spectral peaks between sleep and wake [88]. This model allows viewing the arousal states in terms of a tent diagram with the axes related to stability of the neuronal loops within the corticothalamic system. Different parts of the diagram and hence the stability zones of the system, correspond to stability of different wake and sleep states in the EEG, such as SWA. Alternatively, Weigenand et al. demonstrated generation of SWA in a cortex-only model and proposed an explanation in terms of nonlinear dynamics theory – that the large amplitude of slow oscillations results from a proximity of a homoclinic bifurcation that slows down the oscillation near a stable focus [97].

Majority of the large-scale mean field models of sleep focus on EEG. However, recently a model using and reproducing MRI data has been proposed [98]. This model used fMRI-based connectivity matrix to generate cortical network and simulated BOLD activity at the whole-brain level during sleep and wake. Use of MRI gives an advantage of accessing deeper level brain structures but whole-night MRI recordings for sleep are difficult to obtain due to impracticality of sleep in the scanner. Nevertheless, various sleep-related data are accumulating in the MRI domain, so it is likely that more MRI-based models will be developed in the future.

A limitation of the large-scale mean field models is that they do not consider the conceptual frameworks for sleep regulation (e.g., the two-process model) or the role of sleep-regulatory networks. This is starting to change in the recent years. In 2016 Costa et al. [99] proposed a first model combining cortical and sleep regulatory populations with the homeostatic and circadian processes, which allows generation of the EEG across the sleep-wake and REMS-NREMS cycles and investigation of the role of subthalamic regions in cortical dynamics. Earlier, in 2015, Robinson et al., [85,88] proposed a framework for connecting the corticothalamic neural field model [84] with the mutual inhibition model of the ascending arousal system [100] into a unified “working” brain model but this is yet to be done numerically. 

Overall, the large-scale mean field models of sleep use data from anatomical, electrophysiological and imaging experiments to simulate brain structures responsible for dynamics observed in EEG and MRI. They thus provide a useful tool for testing neurobiological hypotheses, designing tailored experiments and testing effects of lesions or brain stimulation. Another important application of these models is an automatic and continuous tracking of EEG sleep states [86,93]. Traditional sleep staging involves manual scoring of sleep EEG in intervals of 30 s following the guidelines by Rechtschaffen and Kales. This results in a simplified and partially subjective view of the EEG signal categorized into 5 states (N1, N2, N3, REMS, wake), thus losing significant amount of information. Conversely, the models convert EEG into a trajectory in a space of connections within the corticothalamic system and corresponding sleep states [93] thus also providing an insight into the underlying physiology. This opens new avenue for EEG analysis, especially in clinical applications for identifying new EEG markers of disease.

### 3.2. Sleep Regulatory Networks

Models of sleep regulatory networks aim to advance understanding of the roles of specific, mostly sub-thalamic, brain areas in generating behavioural sleep-wake patterns and the NREMS-REMS cycles during sleep [100,101,102,103,104,105]. They focus mainly on the dynamics of the ascending arousal system in the hypothalamus and brainstem and account for the two-process concept by incorporating the effects of the homeostatic and circadian processes, S and C. The two processes are usually modelled as electric current or voltage terms in the equations for respective neuronal populations with S reproducing exponential rise and decay of sleep pressure and C modelled as a sine function. The key brain areas of interest are sleep-active ventrolateral preoptic hypothalamus (VLPO) and wake- and/or REMS active populations such as serotonergic dorsal raphe (DR), histaminergic tuberomammillary nucleus (TMN), noradrenergic locus coeruleus (LC), orexinergic (also called hypocretinergic) lateral hypothalamic area (LHA), cholinergic laterodorsal tegmental-pedunculopontine (LDT/PPT) and glutamatergic basal forebrain (BF) [9,10,106]. The models often organize the different brain areas in populations according to their activity and function: sleep-active, wake-active, REMS-active, REMS-on and REMS-off and introduce coupling among them according to anatomical data and a physiological hypothesis being tested. 

The outputs of these models are time series of populations’ mean voltages and firing rates. The states of wake, NREMS and REMS are identified from the neuronal dynamics of these populations; for example, sleep is reported when sleep-active population demonstrates high level of activity while the wake-active population has low activity. Several neural mass models of sleep-wake cycles were published in the last 15 years [100,101,102,103,104,105,107,108,109,110]. The details of the modelling approaches and differences among them were reviewed in great detail [53]. Examples of models of sleep regulatory networks are given in Table S3.

The simplest of the sleep regulatory network models was proposed by Phillips and Robinson in 2007 [100]. It includes only two populations: one sleep-active and one wake-active, with the switch in activity between them controlled by the homeostatic and circadian drives. It reproduces a large variety of phenomena, including sleep in different mammals [100,111], effects of sleep deprivation [112], effects of caffeine [113], development of narcolepsy [114] and microsleeps [115]. A dynamic circadian oscillator entrained by light [80] was added to the model which enabled the study of chronotypes [116], spontaneous internal desynchrony [117] and circadian phase of sleep propensity [118]. 

A slightly more complicated model, focusing specifically on mouse sleep, was proposed by Diniz Behn et al. It adds a REMS-active population to sleep- and wake-active ones to enable generation of REMS-NREMS cycling [101,119]. The model reproduces both qualitative and quantitative features of mouse sleep and has proposed that orexin plays a key role in generation of sustained wake bouts. This modelling approach was further extended to incorporate explicit neurotransmitter dynamics to study effects of microinjections [108], adjusted to account for human sleep and spontaneous internal desynchrony [120] and simplified to a 1-D map to study relationship between sleep onset, REMS patterning and circadian phase [121]. Another model, initially focusing on mouse sleep but incorporating more physiological components, was proposed by Tamakawa et al. [107]. It incorporates 10 neuron types falling into 4 activity categories (sleep, wake, REMS and REMS+wake) and 2 sleep-promoting substances, thus allowing for probing the roles of the specific brain areas and substances on sleep-wake and REMS-NREMS transitions. 

A number of models took a different approach to incorporating REMS-NREMS cycles by considering REMS-on and REMS-off populations instead of a single REMS-active population, the so-called mutual inhibition hypothesis versus a reciprocal interaction one [53]. As early as 1974 Feinberg proposed a conceptual model of interaction between NREMS and REMS across the sleep cycles [122]. McCarley and Hobson proposed a first mathematical model for NREMS-REMS regulation based on the mutual inhibition between REMS-on and REMS-off populations [123,124,125,126]. Several modern models, including that by Rempe et al. and Kumar et al., follow this approach, incorporating at least four types of neuronal populations: sleep, wake, REMS-on and REMS-off and considering a more detailed REMS-NREMS switch [102,103,104]. For detailed reviews of NREMS-REMS cycling modelling see [53,127]. 

Several sleep regulatory networks models focused on roles of specific neuron types and neurotransmitters in microstructure of sleep and wake [105,109,110]. These models mainly operate at the seconds, rather than days, timescale and thus do not account for the homeostatic or circadian drives. For example, Patel and Rangan focused on the role of LC in distribution of the wake bouts and showed how it allows to shift the distribution from exponential to power law during development in rats [105]. Mosqueiro et al. proposed the role of LC, fast amino acid and a slow neuropeptide in generation of wake-NREMS transitions [109] and Jalewa et al. studied the different timescales of interaction between orexin and serotonin neurons [110]. In functionality, these models are similar to the cellular level models in Section 4.2. but incorporated with a neural mass approach allowing for faster computation while considering averaged dynamics.

Neural mass models of sleep regulatory networks provide an important link between sleep neurobiology and behavioural sleep-wake patterns. So far, they are unique in this property, because the neural mass approach allows for detailed simulation of mean neuronal and neurotransmitter activity while being computationally efficient, so that dynamics at the timescale of days, weeks and even years can be simulated. The key uses of these models are to (i) integrate diverse electrophysiological and behavioural experimental data into theories of sleep regulation, (ii) provide a tool for testing new hypotheses and (iii) predict sleep dynamics under different conditions. The limitation, however, is that they do not allow access to endogenous neuronal dynamics, specific spiking patterns and configurations of neuronal networks at a cellular level. 

## 4. Neuronal Interactions—Cellular Level

Models at the cellular level aim to understand the role of specific neurotransmitters, neuromodulators, ion channels, spiking patterns and cell types in regulation of sleep and in generation of brain dynamics. Opposed to models at the neuronal population level, these models simulate details of individual neurons and synaptic connections thus allowing the study of the interplay among the endogenous neuronal dynamics, coupling with other neurons and their effects on larger scale dynamics. They mostly use the so-called conductance-based or Hodgkin-Huxley type modelling approach, where ionic currents across the neuronal membrane are modelled explicitly according to their experimentally recorded voltage- and transmitter-dependent activation properties [128]. Depending on a problem, these models include anywhere between one and tens of thousands of interacting neurons. The outputs of these models are time series of voltages and ionic currents of individual neurons and simulations are compared to electrophysiological recordings either from single cells or, cell groups. The variables of these models are the voltages and activation rates of ionic currents with parameter values often directly taken from experimental measurements. Similar to the mean field level, models at the cellular level can be divided into those focusing on the large-scale brain dynamics of the cortex and thalamus (Section 4.1) and the sleep regulatory networks (Section 4.2).

### 4.1. Large-Scale Brain Dynamics—Cortex and Thalamus

Cellular models of large-scale brain dynamics allow investigation of firing patterns, synchronization and effects of specific ion channels and synapses on EEG features. Similar to the mean field models of large-scale dynamics, cellular models focus on the thalamocortical (corticothalamic) system and its components and do not account for the circadian and homeostatic interaction. A number of them, however, propose and test mechanisms for the homeostatic process. A summary of large-scale cellular models is given in Table S4. 

Bazhenov et al. proposed a model of thalamocortical circuit based on the neuroanatomy of individual neurons in the thalamus and cortex and connections between them [129]. The model contains hundreds of neurons and reproduces neuronal dynamics associated with SWS and wake, including synchronized bursting activity and asynchronous tonic firing [5]. It was further extended to study the mechanisms of spindles generation and propagation [130,131], mechanisms of memory consolidation due to a combined effect of SWS and spindles [132,133] and mechanisms of sleep stages generation by an interplay between cellular and chemical processes [134]. 

Hill & Tononi proposed a larger model of the thalamocortical circuit with >65,000 neurons which explained the ionic mechanisms behind SWS generation [135] and was used to test the synaptic homeostasis hypothesis of sleep [26]. It shows that decrease in synaptic strength is sufficient to account for changes in SWA [136,137]. Incorporation of spike-time dependent plasticity rules and an interplay of plasticity with neuronal activity in the model allows for self-limiting renormalization of synaptic strength reflecting sleep homeostasis [138]. The mechanisms of memory consolidation during sleep were further tested in a hierarchical neuronal network with the view of translation of the model to hardware devices [139,140].

The local sleep hypothesis [141] was also tested using a cellular level, albeit abstract, model of the cortex [142]. The hypothesis is that sleep is initiated locally in cortical columns in a state-dependent manner; that is, the areas that were used more during prior wakefulness demonstrate higher SWA during sleep. The model demonstrates that the interaction among the simulated cortical columns is critical for the rapid transition to global sleep state. Another cortical model, simulating interaction of 66 cortical areas with 200 neurons each, has demonstrated that a moderate decrease in acetylcholine allows for a transition from wake to SWA in local networks and a larger decrease leads to global sleep across the entire modelled tissue [143]. 

The role of K^+^ leak currents in triggering a transition between wake and sleep and regulation of sleep duration was predicted in several models of different architecture, including both large networks of tens of thousands of neurons and simpler single neuron models [135,144,145,146]. Persistent Na^-^ currents were shown to initiate up-state of the slow oscillations [135] and a hyperpolarization-activated current was predicted to be responsible for spindle oscillations [147,148]. 

Due to the high computational cost of cellular models, new approaches to account for the intrinsic and network dynamics of neurons while reducing computation time are being explored. For example, Komarov et al. proposed a map-based neuron model to simulate cortical dynamics during sleep [149] and Tatsuki et al. considered an average cortical neuron for testing the effects of ionic currents on sleep- and wake-related patterns of activity [144]. Similarly, Paul et al., [150] and Holmgren Hopkins et al. [151] investigated thalamic dynamics by using just 3 and 4 neurons respectively. They demonstrated that the transition from asynchronous tonic firing to synchronized bursting is associated with the reduction of wake-promoting input from the hypothalamus [151,152] and predicted existence of chaotic neuronal dynamics at the transitions between sleep and wake [150,151]. 

The large-scale cellular models of the thalamocortical, cortical and thalamic systems are particularly important for understanding the roles of specific ion channels and synchronization dynamics on higher level brain dynamics observed in EEG. They reconcile electrophysiological patch-clamp experiments, multi-unit recordings and the mean field dynamics of neurons. They can be used to directly simulate blocking/activation of specific ion channels or application of chemicals without a-priory knowledge of their effects on neuronal dynamics. Synchronization properties can be studied in great detail along with the ways to manipulate synchronization and spiking activities. The limitation, however, is that the circadian effects are not considered, largely due to the high computational cost of this models.

### 4.2. Sleep Regulatory Networks 

Similar to modelling of sleep regulatory networks at the mean field level, models at the cellular level focus on dynamics of the neurons of the ascending arousal system in the hypothalamus and brainstem. These models are aimed at understanding specific features of the system or experimental observations rather than reproducing sleep-wake patterns. Examples of models at this level are given in Table S5.

There are only several models at this level and all of them focus on dynamics of the neuropeptide orexin and orexin neurons in the lateral hypothalamic area [153,154]. Postnova et al. proposed a mechanism for sleep homeostasis based on the synaptic plasticity of orexin projections and demonstrated it with 2 coupled orexin and glutamate neurons [155]. Sleep-wake cycles were simulated with the addition of a skewed sinusoid circadian drive, implemented as an external current input to the orexin neuron. The model was later used to study effects of hypothalamic drive on thalamic neurons [151] and extended to incorporate neuronal diversity and noise demonstrating that diversity in orexin neurons is beneficial for stability of the sleep-wake cycle [156]. Carter et al. used experimental and modelling approaches and demonstrated that orexin action on sleep-wake transition is gated by the LC neurons and that the orexin-LC circuitry is critical in integration of orexin effects [157]. The model was extended to simulate the effects of optogenetic stimulation of orexin neurons on the LC neurons [158] and to investigate the role of GABA in the orexin-LC circuit. It has predicted that a slow, yet unidentified, inhibitory neuropeptide is likely involved in the neuromodulation of the circuit [109]. Williams and Diniz Behn used a two-neuron model to investigate the delay in functional orexin neurons effects at the transition from sleep to wake and predicted that it is due to the desensitization of orexin neurons to dynorphin [159].

Cellular models of sleep regulatory networks are important for understanding the detailed mechanisms controlling sleep-wake transitions at the microscale level in both time and space. Unlike the neural mass models, cellular models do not consider the sleep regulatory system as a whole and, with one exception [155], do not consider the circadian or homeostatic processes. These are designed to answer specific questions arising from experiments and are often developed along with experimental studies as a complementary approach [109,157,158,159].

## 5. Discussion

Models of sleep regulation vary in the physiological level they focus on, the part of the sleep system they address and methodology used. The physiological levels of the existing models are: behavioural, level of interacting brain areas (mean field) and level of interacting neurons (cellular). There appear to be no models at the molecular level, which is largely due to that fact that the genetic and molecular mechanisms of sleep are not yet studied sufficiently for developing a theory. This is opposed to the modelling of circadian rhythms, where majority of the models are at the molecular level and the genetic machinery of the clock is well established [44,45,46,47,48,49,50,55,56,57,58,59]. The brain parts typically addressed are the thalamocortical system and the sleep regulatory networks in the hypothalamus, brainstem and forebrain. Methodology varies from abstract functions to describe qualitative dynamics, to neural field allowing for waves’ propagation in both time and space and to conductance-based modelling with details of ionic currents and neurotransmitters (see Tables S1–S5 for examples). Approaches combining/modifying these to allow establishing links across the levels have also been presented [83,108,109,144,145,149].

Most of the models focus on one level of physiological functioning. Behavioural level models are important in understanding the big picture of sleep regulation and providing a high-level framework for explanation of detailed mechanisms at “lower” levels. The mean field models allow for better understanding of the brain areas involved and their interaction, while the cellular level models focus on details of ionic dynamics and explain how and why neuronal activity changes. Importantly, many of the mean field and cellular level models of cortex and thalamus are generic models of these brain structures and address many different phenomena, including seizures, epilepsy, Parkinson’s disease and evoked response potentials [160,161,162,163]. Conversely, models of sleep regulatory networks are built to explain sleep dynamics and their applications are currently limited to sleep-related phenomena. 

The dynamics at lower levels of molecular and neuronal interactions underlie the activity of neuronal populations and our behaviour. The level of complexity and the number of contributing factors are increasing dramatically as one moves to lower physiological levels. While at the behavioural level we focus on 2 processes, at the cellular level one has to consider a variety of neurotransmitters, neuromodulators, ionic currents, neuronal firing activity and coupling among neurons. Understanding connections across these levels as well as within the levels is needed for developing an integrated system theory of sleep. Models have already started to address some of the connections across the levels as discussed in more detail below. 

### 5.1. Links between Cellular and Behavioral Levels 

Majority of the models at cellular level do not account for behavioural sleep-wake patterns or circadian variation. Instead they simulate effects of sleep by adjusting model inputs or parameters. The main obstacle is the difference in time scales of neuronal dynamics (milliseconds) and the daily rhythms (24 h) [164]. On one hand, this allows us to simplify models by assuming that the long time-scales do not affect the dynamics on short time-scales and can thus be neglected. On the other, if the aim is to study the cross-scale interaction, it makes it difficult to do so mathematically and requires large computation times. For example, models with networks of conductance-based neurons require minutes (sometimes hours) of computer time to simulate 1 second of real time. This translates into weeks of simulation time for 1 sleep-wake cycle and is prohibitive for study of behavioural sleep patterns. Several models, however, have tried to address this by simulating just 1–4 neurons [144,145,151,155] and scaling of the 24 h cycles to 24 seconds [151,155]. By using just 1 “averaged” neuron, Yoshida et al. [145] predicted the effect of K^+^ leak current on sleep duration and then confirmed it experimentally. 

For similar reasons, cellular level models do not account for the homeostatic sleep process, except for the ones proposing and testing new hypotheses for the mechanisms of sleep homeostasis [94,136,138,142,155]. These predominantly focused on synaptic plasticity of neuronal connections. It is possible, however, that sleep homeostasis is a multi-level process and different mechanisms contribute across the levels. 

### 5.2. Links between Cellular and Mean Field Levels

In theory, cellular models can be directly linked to mean field dynamics by averaging activity across the cells. In practice, however, this can be true only for models with very large number of cells to simulate a piece of a tissue from which a mean field level recording is made. This is potentially the case for large network models of the thalamocortical system, like that of Hill and Tononi with >65,000 neurons [135] or smaller ones with about 1000 neurons [8,129,139,140,146]. Even these models are small compared to the actual brain size of about 100 billion neurons. However, the utility of further increasing the number of modelled neurons for understanding sleep dynamics is unclear while the computational cost increases significantly with the size of the network. A different way of linking the cellular and mean field levels may thus be of interest—e.g., by using hybrid models, like that of Deco et al. [143]. This model simulates 66 cortical nodes with 200 neurons in each with the connections among the nodes provided by fMRI connectivity matrix. It thus benefits from mean field data and allows access to dynamics and manipulation of individual neurons at the same time (the neurons are simplified, however). 

Cellular models of sleep regulatory networks tend to focus on specific mechanisms of sleep and sleep-wake transitions and do not incorporate the entire ascending arousal system like the neural mass models do. So far, these models mainly focused on the orexin-related dynamics and included relatively small number of neurons—from 2 to 60 [109,155,156,157,158,159]. Depending on the problem being solved, both cellular and mean field approaches may be used. For example, Mosqueiro et al. have developed a model of orexin-LC-GABA circuit at both neuronal and neural mass levels to first test the mechanisms at the level of ionic currents and then examine how they translate to population dynamics [109]. Alternatively, Diniz Behn and Booth developed a novel modelling framework incorporating details of neurotransmitter concentrations at the level of synapses into a neural mass model thus addressing elements of dynamics at both levels and allowing to simulate microinjections [108].

### 5.3. Links between Mean Field and Behavior Levels

Mean field level models (Section 3) are best equipped to provide links with behavioural sleep-wake cycles level due to their lower computational requirements. The mean field cortical and thalamocortical models (Section 3.1) focus on the millisecond scale dynamics, similar to the cellular models. However, the number of interacting units and equations is significantly lower than in the cellular models (e.g., 4 compared to 65,000), thus allowing for faster simulations. However, in order to reproduce sleep-wake cycles these models require input from either the sleep regulatory networks or the two-process model. Recently, several attempts were made to connect mean field EEG and sleep regulatory models to allow for investigation of the links between the ascending arousal system and thalamocortical system in generation of EEG and sleep-wake behaviour [85,99]. Costa et al., in particular, combined the neural mass sleep regulatory model by Diniz Behn et al. [101] with the cortical model by Weigenand et al. [97] thus enabling simulation of 24-h sleep-wake cycles and study of the links between different sleep regulatory populations and the EEG. 

Mean field models of sleep regulatory networks provide a direct link between neuronal dynamics of the ascending arousal system and sleep-wake behaviour. They also incorporate the homeostatic and circadian processes as proposed by the two-process model. This link can be easily made because these models focus on differences in mean neuronal dynamics between the states of wake, NREMS and REMS but not the details of dynamics within each of these states. Thus they operate on the scale of minutes rather than milliseconds; for example, the model by Diniz Behn et al. requires a simulation time step of 600 ms [101] compared to 0.1 ms in the combined EEG and sleep model by Costa et al. [99] and 0.01 ms in the cellular thalamocortical model by Bazhenov et al [129]. 

Many of the mean field sleep network models use Morris-Lecar type equations, which are a simplification of the Hodgkin-Huxley type neuron model [128]. This allows for oscillatory dynamics of individual populations and incorporation of different ionic currents [101,102,103]. Phillips et al., used a different approach, assuming that in absence of external inputs a neuronal population would have constant mean voltage level of 0 mV. This allowed for faster simulations, so that only a few seconds of computer time are needed to simulate a week of real time. This made this model very attractive for studying real world applications where a simulation of a large number of days is of interest, such as shift work [165,166,167], jetlag [85], effects of ageing [168,169] and prediction of alertness [71,170]. 

### 5.4. Future Directions

Models at the different physiological levels of sleep regulation and of the different relevant brain systems have contributed significantly to our understanding of sleep. Further development of models at the different physiological levels will be important for integration of experimental findings and explaining the mechanisms. As discussed above, models attempting to connect several levels are starting to be developed [99,108,142,143,144,145,149,151] and all of the neural mass models of sleep regulatory networks already span across neuronal populations and behavioural levels (see Section 3.2). Further development of these links will be important for understanding the complex system of sleep regulation as a whole. 

Other components of the system at the different levels require more attention as well. For example, the interaction between neurons and astrocytes was shown to be involved in metabolite clearance during sleep [171] and in regulation of sleep duration [172] but is yet to be considered in any of the models. Several chemicals and neurotransmitters have been identified as candidate contributors to the homeostatic sleep process but the interaction among them is unclear and is yet to be reconciled in a unified theory [28]. Molecular level models of sleep, both conceptual and mathematical, are missing as well. Before such models can be developed, however, more experimental research is needed to advance our understanding of genetic and biochemical mechanisms of sleep regulation. 

Integration of the circadian clocks with the sleep regulatory mechanisms will be another important direction. Current models of sleep either incorporate phenomenological dynamic circadian oscillator for example, [116,117,120,173], simulate circadian process with a sine function for example, [12,100,101,102,103,107,151] or do not consider it at all for example, [94,97,129,135,143]. While this is sufficient for many applications it does not advance our understanding of the interaction between the circadian and homeostatic mechanisms. With the increasing amount of evidence pointing to complex interdependencies between the two processes across physiological levels [35,36,37] it will be beneficial to develop models that explain the mechanisms. 

## Supplementary Materials

The following are available online at https://www.mdpi.com/2624-5175/1/1/15/s1. [174,175,176]. Table S1: Examples of mathematical models at the behavioural level of physiological functioning. Table S2: Examples of mathematical models at the large-scale mean field level. Table S3: Examples of models at the sleep regulatory mean field level. Table S4: Examples of models at the large-scale cellular level. Table S5: Examples of mathematical models at the cellular sleep regulatory level.

## Abbreviations

EEG	Electroencephalography
SWA	Slow wave activity
ipRGC	Intrinsic photosensitive retinal ganglion cell
SWS	Slow wave sleep
REMS	Rapid eye movement sleep
NREMS	Non-rapid eye movement sleep
SCN	Suprachiasmatic nucleus
fMRI	Functional magnetic resonance imaging
BOLD	Blood oxygen level dependent imaging
