# Supplementary material for: Sleep Modelling across Physiological Levels
Source: Clocks Sleep. 2019 Mar 4;1(1):166–84. doi: 10.3390/clockssleep1010015 (PMC7509680; doi:10.3390/clockssleep1010015)
Supplement: Supplementary file 1 [file clockssleep-01-00015-s001.pdf]

clocks & sleep

Supplementary material

Sleep Modelling across Physiological Levels

Svetlana Postnova [1,2,3]

[1] School of Physics, University of Sydney, 2006 NSW, Australia; E-Mail: spostnova@sydney.edu.au
[2] Center of Excellence for Integrative Brain Function, University of Sydney, 2006 NSW, Australia
[3] Charles Perkins Center, University of Sydney, 2006 NSW, Australia

Received: 20 December 2018; Accepted: 25 February 2019; Published: 4 March 2019

Table S1. Examples of mathematical models at the behavioral level of physiological functioning. The models are listed in a chronological order and studies using the same core model are grouped.

	**Behavioral level**	
**Study**	**Key features**	**Approach**
Kawato et al., 1982 [77]	Focused on the circadian rhythm of sleep. Modelled free running state of the human circadian rhythm as marked by the body core temperature. Simulated sleep duration, irregular free-running pattern, wake maintenance zone, and internal desynchronization.	3 coupled oscillators and a sine function for the circadian clock
Kronauer et al., 1982 [78]	Focused on the circadian rhythm of sleep. Simulated circadian sleep duration, the transition from normal sleep activity to internal desynchronization, and misalignment of sleep and temperature after jetlag.	2 coupled oscillators with a cosine zeitgeber input to one
Daan et al., 1984; Daan & Beersma 1984 [12,64]	Proposed two-process model of sleep, where sleep timing is determined by interaction between the circadian (C) and homeostatic (S) processes. The S process was based on SWA in sleep EEG. Simulated internal desynchronization, sleep fragmentation, sleep propensity, sleep rebound after sleep deprivation and sleep pattern during shift work.	Exponential rise and decay of S during wake and seep respectively and 2 circadian skewed sine thresholds
Achermann & Borbely 1990 [65]	Adjusted the original model so that the change of S rather than S itself were proportional to SWA and added ultradian rhythm for REMS-NREMS cycles.	
Franken et al., 1991 [67]	Adjusted the two-process model for rat sleep. Proposed a quantitative version of the model.	
Achermann et al., 1993 [66]	Calibrated the process S using large number of PSG recordings.	
Putilov 1995 [68]	Introduced circadian variation of the homeostatic process.	
Strogatz 1987 [79]	Focused on the circadian rhythm of sleep. Simulated internal desynchronization of the sleep/wake rhythms and core body temperature.	2 coupled oscillators with a cosine input
Nakao et al., 1995; Nakao et al., 2007 [54,82]	Proposed that sleep homeostasis is controlled by thermoregulatory mechanisms. Simulated core temperature rhythm, sleep patterns, and effect of sleep deprivation.	2 circadian oscillators and 2 feedback loops for temperature regulation
Bes et al., 2009 [73]	Introduced a new circadian sleep drive R which was based on REM latency data. Modeled sleep propensity as a continuous	Based on the two-process model

	variable over 24 h. Sleep propensity modelled as a product of S and R. Simulated sleep propensity, napping, and post-lunch dip.	
Schmidt et al., 2017 [83]	Focused on energy allocation during sleep and wake. Predicted that energy savings derived from sleep may be 4-fold greater than previous estimates.	3 oscillators driven by a sine circadian function

Table S2: Examples of mathematical models of cortex and thalamus at the mean field level.

**Mean field level – cortex and thalamus**		
**Study**	**Key features**	**Approach**
Steyn-Ross et al., 2005 [96]	Simulated cortical dynamics resulting in EEG features of SWS and REMS. Proposed that the transition from SWS to REMS can be understood as a phase transition from low-firing synchronized state to high-firing asynchronous dynamics.	Neural field theory; 2 cortical populations
Robinson et al., 2005,2011,2015 [84,85,88]	Simulated the dynamics of the corticothalamic system and generation of EEG dynamics. Reproduced key features of wake and sleep EEG power spectrum.	Neural field theory; 4 populations: 2 cortical and 2 thalamic
Abeysuriya et al., 2014,2014 [90,91]	Demonstrated that nonlinearity in the thalamic relay nuclei induces spindle harmonic in EEG. Predicted and verified that the power in the spindle harmonic scales quadratically with the power in the spindle oscillation.	
Zhao et al., 2015 [89]	Incorporated bursting dynamics in the thalamic nuclei. Reproduced cortical "up" and "down" states in deep sleep and other EEG spectral features of sleep.	
Abeysuriya et al., 2015, 2016 [92,93]	Identified connections within the corticothalamic model that are responsible for generation of different sleep states and proposed a way of continuous tracking of arousal state as opposed to the discrete Rechtschaffen and Kales sleep staging.	
Assadzadeh & Robinson 2018 [94]	Demonstrated necessity for sleep-wake cycles to maintain synaptic plasticity in the cortex.	
Weigenand et al., 2014 [97]	Simulated interaction between populations of cortical neurons to generate EEG time series and spectra. Described bifurcation structure of the model in relation to slow oscillations and K-complexes.	Neural mass model; 2 cortical populations
Cona et al., 2014 [87]	Simulated interaction between thalamic nuclei and a cortical column consisting of four connected populations. Proposed mechanisms for generation of EEG activity at different frequencies.	Neural mass model; 6 populations: 4 cortical and 2 thalamic
Costa, Born et al., 2016 [99]	Modelled interaction between the sleep regulating areas in the hypothalamus and brainstem and the cortical neuronal populations responsible for EEG. Reproduced EEG features across arousal states over 24 h. Provided a link between EEG-focused models and models of sleep regulatory networks.	Neural mass model; 5 populations: 2 cortical, NREM, REM, and wake
Costa, Weigenand et al., 2016 [95]	Modelled thalamocortical system involved in generation of the EEG. Reproduced EEG features of fast sleep spindles, slow oscillations, K-complexes, and response to auditory stimuli.	Neural mass model; 4 populations: 2 cortical and 2 thalamic
Yamaguchi et al., 2018 [86]	Combined a model of the corticothalamic loop with a stochastic model to track the strength of connections between neuronal populations along the sleep stages and to continuously track sleep-wake transitions.	Neural field theory and an autoregressive model
Deco et al., 2018 [98]	Simulated BOLD activity at the whole-brain level. Simulated the brain in wake and deep sleep state by using BOLD data-derived connectivity matrices. Showed that waking brain operates further from a stable	Abstract; 90 cortical areas with 2 oscillators

	equilibrium than deep sleep. Developed a new tool to study effects of perturbation on the brain in different states.	each and coupled via an fMRI-derived matrix

Table S3: Examples of models of the sleep regulatory networks at the mean field level.

Mean field level - sleep regulatory networks		
**Study**	**Key features**	**Approach**
McCarley & Hobson 1975; McCarley & Massaquoi 1986 [123,124]	Proposed a mutual inhibition model for REM-NREM sleep regulation based on the concept of REM-on and REM-off neuronal populations in the ponine gigantocellular tegmental field (FTG) and locus coeruleus (LC).	Lotka-Volterra equations [123]; later updated to limit cycle[124]; 2 neuronal populations
Massaquoi & McCarley 1992 [126]	Combined the reciprocal REM-NREM sleep model with the two-process model.	
McCarley & Massaquoi 1992 [125]	Extended the model by adding the cholinergic system for regulation of REM sleep and SWA.	
Tamakawa et al., 2006 [107]	Proposed a quartet neural system model of four interacting populations: sleep-active, wake-active, REM-active, and wake+REM-active; and 2 sleep-promoting substances. Reproduced sleep and wake patterns in rats and the sleep-related neuronal activities. Human sleep-wake patters were simulated by adjustment of parameters.	Abstract neural mass; 10 neurons, 4 populations
Phillips & Robinson 2007, 2008; Phillips, Robinson et al., 2010 [100,112,174]	Modelled the ascending arousal system (AAS) composed of mutually inhibitory sleep-active monoaminergic neurons in the hypothalamus and brainstem and wake-active neurons in the VLPO of the hypothalamus [9]. Reproduced mammalian sleep patterns, effect of sleep deprivation on sleep duration, and proposed existence of hysteresis at the transitions between sleep and wake.	Neural mass; 3 populations [114]; 2 populations otherwise
Puckeridge et al., 2011 [113]	Incorporated effects of caffeine on sleep dynamics and fatigue.	
Phillips, Chen et al., 2010; Phillips et al., 2011 [116,117]	Combined the AAS model with the dynamics circadian oscillator [80]. Reproduced spontaneous internal desynchrony as a result of changes in orexin input and dynamics of different chronotypes. Enabled simulations of shiftwork and jetlag.	
Phillips et al., 2013 [175]	Incorporated REMS-NREMS cycling dynamics during sleep. Proposed that ultradian cycling develops due to the arousal state feedback to the circadian and homeostatic processes. Reproduced key NREMS-REMS patterns and NREM rebound after deprivation.	
Fulcher et al., 2014 [114]	Incorporated orexin population as the regulator of stability of wake and sleep. Showed development of narcolepsy due to reduced orexin effects.	
Yang et al., 2016 [115]	Incorporated noise in the AAS model. Proposed dynamical precursors of sleep near wake to sleep transition and of microsleeps.	
Postnova et al., 2016 [118]	Modified circadian input to AAS to reproduce ciradian phase dependence of the sleep propensity. Reproduced forced desynchrony data for total sleep duration and sleep latency.	
Diniz Behn et al., 2007,2008 [101,119]	Modelled network dynamics governing mouse sleep-wake behavior consisting of wake-, sleep-, and REM-active populations. Reproduced qualitative and quantitative features of mouse sleep, including REM-	Neural mass, Moris-Lecar type; 3 populations

	NREM cycling. Demonstrated the difference between brief and sustained awakenings and proposed that these dynamics are regulated by orexin.	
Diniz Behn & Booth 2010 [108]	Incorporated neurotransmitter dynamics in the model to simulate effects of microinjections. Reproduced effects of GABAergiic and cholinergic agonists/antagonists on the rat sleep-wake behaviour.	
Gleit et al., 2013 [120]	Modified the model for human sleep and combined with the dynamics circadian oscillator [81]. Predicted that the strength of SCN projections can account for individual variability in spontaneous internal desynchrony. Demonstrated the effects of internal desynchrony on REMS-NREMS cycles.	
Booth et al., 2017 [121]	Developed a one-dimentional map of sleep onset and REM patterning depending on circadian phase based on the mouse sleep model [101].	
Rempe et al., 2010 [102]	Simulated the flip-flop switch between the sleep- and wake-active neurons in the hypothalamus and brainstem including sleep- and wake-active populations, orexin, and REM-on, REM-off populations. Reproduced the timing of sleep, effects of sleep deprivation, ultradian rhythms, development of narcolepsy, and phase-dependence of total sleep time.	Neural mass; Moris-Lecar type; 4 populations.
Kumar et al., 2012 [103]	Simulated sleep-regulatory mechanisms for NREMS-REMS cycle. Reproduced key sleep patterns, including the effect of sleep deprivation and phase-dependence of sleep onset. Confirmed that REMS is generated via pre-synaptic inhibition of REM-off terminals that project on REM-on neurons. Proposed that orexin neurons act as gatekeeper to balance the inputs from the circadian clock and from REMS circuits.	Neural mass approach, Moris-Lecar type; 6 populations
Dunmyre et al., 2014 [104]	Constructed single flip-flop and coupled flip-flop models based on population firing rate and neurotransmitter formalism [108]. Showed that transitions from REMS to wake require excitatory input from REM-on to the wake population. Wake to NREMS transitions required modulation of REM-on and REM-off populations by the wake population.	Neural mass with explicit neurotransmitter dynamics; 2 populations for single and 4 for coupled flip-flops
Mosqueiro et al., 2014 [109]	Modelled hypocretin – locus ceruleus circuitry including the effects of GABA and slow inhibitory neuropeptide that was proposed to be responsible for control of wake-NREMS transitions. The model also has a conductance-based version.	Neural mass; Wilson-Cowan equations; 4 populations
Jalewa et al., 2014 [110]	Simulated interaction between the orexinergic population in the lateral hypothalamus and serotonergic population in the dorsal raphe to explore the effects of connections and time scales on dynamics. Showed that at fast time scale orexin activation can lead to increase in activity of serotonergic neurons.	Neural mass; 4 populations
Patel & Rangan 2017 [105]	Modelled the interaction among the sleep-, wake-active populations in the hypothalamus and brainstem and the locus coeruleus (LC) to study developmental changes of sleep patterns in rats. Showed that change of physiology of LC during development along with reciprocal excitation betweem LC and wake population explains the shift from exponential to power law distribution of the wake bouts.	Neural mass; Wilson-Cowan equations; 3 populations

Table S4: Examples of models of the cortex and thalamus at the cellular level.

	**Cellular level – cortex and thalamus**	
**Study**	**Key features**	**Approach**
Destexhe et al., 1994 [148]	Modeled reticular thalamic nuclei (RE) isolated from other influences. Demonstrated that an interplay between intrinsic dynamics of individual neurons and fast synaptic interactions are required to reproduce spindles as observed in sleep stage 2.	A network of Hodgkin-Huxley type neurons [128]; varied number of neurons, up to 1,600; 1 thalamic population [148] and 2 thalamic populations [147]
Destexhe et al., 1996 [147]	Added thalamocortical (TC) neurons for a more complete simulation of thalamus. Proposed that the activity dependent upregulation of hyperpolarization-activated I_h current in TC cells and localized projections between TC and RE are responsible for the initiation, formation, and propagation of spindle oscillations.	
Bazhenov et al., 2002 [129]	Modelled thalamocortical system with detailed interaction among individual neurons. Proposed that cortical slow waves are generated via the "re- excitation" of the cortical network on each cycle of oscillation by the spontaneously occurring coinciding miniature EPSPs.	A network of Hodgkin-Huxley type cortical and thalamic neurons coupled via transmitter-gated synapses. 175 neurons with hundreds of connections in 4 populations: 2 cortical and 2 thalamic
Bonjean et al., 2011, 2012 [130,131]	Demonstrated that the propagation of spindles depends on synaptic interaction within the thalamus, but the initiation and termination of spindles involved the corticothalamic network.	
Wei et al., 2018 [132,133]	Demonstrated that interaction between hippocampal input, cortical slow oscillations and synaptic plasticity lead to consolidation of memories through replay of cortical cell spike sequences during SWS. Showed that sleep spindles and SWS both promote memory consolidation.	
Krishnan et al., 2016 [134]	Identified critical intrinsic ionic and synaptic mechanisms resulting in characteristic EEG features of vigilance states and transitions between sleep stages.	
Compte et al., 2003 [146]	Modelled cortical dynamics with a network of interacting neurons. Reproduced experimentally observed slow rhythmic activity (<1Hz). Demonstrated that spontaneous spikes in single neurons are sufficient to trigger waves of activity propagating across cortical tissue. Predicted a bi-stability of up- and down-states in the cortex. Showed that manipulation of leakage K^+ currents lead to a transition to slow oscillations.	A network of Pinsky-Rinzel [176] and Hodgkin-Huxley type neurons. 1,290 neurons with random connections.
Hill & Tononi 2005 [135]	Modelled thalamocortical system at the neuronal level by simulating networks of coupled neurons following detailed anatomy. Determined mechanisms responsible for dynamics of up-states during deep sleep, including that an increase in leak K^+ current is sufficient to trigger the transition from wake to sleep, the up-state of the slow oscillation can be initiated by activation of persistent Na currents, and corticocortical connections synchronize slow oscillations.	A network of Hodgkin-Huxley type neurons coupled via transmitter-gated synapses; >65,000 neurons and millions of connections in 4 populations: 2 in visual cortex and 2 in thalamus
Tononi & Cirelli 2006; Esser et al., 2007 [26,136]	Proposed the synaptic homeostasis hypothesis (SHY); i.e., that synaptic strength increases during wake and SWA serves to downscale it. Simulated reduction in synaptic strength in the neuronal thalamocortical system model [135] and showed that this decrease is sufficient to account for changes in sleep SWA	

Olcese et al., 2010 [138]	suggesting a link between the homeostatic process S and the synaptic strength. Incorporated spike-timing dependent learning rules in the model and showed that the interplay between plasticity and neuronal activity results in self-limiting renormalization of synaptic strength reflecting sleep homeostasis properties.	
Hoel et al., 2016 [137]	Demonstrated that synaptic refinement can account for developmental changes in SWA, and in particular for SWA drop during adolescence.	
Krueger et al., 2008; Roy et al., 2008 [141,142]	Proposed the activity-dependent theory for sleep; i.e., that a whole organism sleep emerges from the local sleep states of cortical columns in an activity-dependent manner. Demonstrated these dynamics in an abstract model of interacting cortical columns and proposed that the interaction among columns is critical to the rapid formation of a global sleep state.	Abstract; Activity-Integrator Network
Nere et al., 2012,2013 [139,140]	Used a network of coupled integrate-and-fire neurons with plasticity to show that synaptic down-selection during sleep is sufficient to explain the beneficial effects of sleep on memory consolidation.	Hierarchical network of integrate-and-fire neurons; 766 neurons
Deco et al., 2014 [143]	Modelled cortical network according to its anatomical connectivity to evaluate functional connectivity when the cortex falls asleep. Demonstrated that moderate decrease in ACh allows for a transition to SWA in local networks, and stronger decrease led to global SWA across the entire cortex. The connection matrix in the model is derived from fMRI data.	Network of 66 coupled attractors represented by 200 integrate and fire neurons; 2 cortical populations
Paul et al., 2016 [150]	Reproduced neuronal oscillatory activity related to sleep and wake in simplified thalamocortical model. Demonstrated chaotic dynamics at the transitions between sleep and wake.	Hodgkin-Huxley type; 3 neurons
Tatsuki et al., 2016 [144]	Developed a cortical averaged neuron (AN) model to study effects of specific ion channels on SWA. Predicted that Ca^{2+}-dependent pathway plays a role in regulation and duration of sleep. Confirmed this experimentally in KO mice.	Hodgkin-Huxley type; 1 "averaged" cortical neuron based on neurons in thalamocortical network models [129,135]
Yoshida et al., 2018 [145]	Simplified the AN model to identify redundant components. Predicted that K^+ leak channels are responsible for regulation of sleep duration. Confirmed this experimentally in KO mice.	
Komarov et al., 2018 [149]	Introduced a new class of models to simulate cortical dynamics during sleep and wake. Predicted critical role of synaptic noise in memory consolidation.	Map-based neuron model
Holmgren Hopkins et al., 2018, Postnova et al., 2011 [151,152]	Simulated dynamics of gap junction coupled thalamic neurons driven by wake-active neuronal populations in the hypothalamus. Demonstrated that wake active neurons can drive tonic to bursting and asynchrony to synchrony transitions associated with wake and sleep in the thalamus. Showed that noise is essential for asynchronous firing in wake and that the same noise does not perturb synchronous bursting in sleep. Predicted presence of chaotic neuronal firing at the transitions between sleep and wake.	Hodgkin-Huxley type neurons; 4 coupled neurons

Table S5: Examples of mathematical models at the cellular sleep regulatory level.

Cellular level – sleep regulatory networks		
**Study**	**Key features**	**Mathematical formulation**
Postnova et al., 2009 [155]	Proposed that synaptic plasticity of orexin neurons is involved in the homeostatic regulation of sleep. Reproduced firing activity of orexin and glutamate neurons during sleep-wake cycles, effects of sleep deprivation and napping on duration of following sleep. Showed the delay between the onset of orexin neurons firing and activation of connected neurons.	Hodgkin-Hyxley type neurons coupled via transmitter-gated and gap-junction synapses; 2 neurons [155], 21 neurons [156]
Patriarca et al. 2012 [156]	Extended the model to simulate networks of reciprocally connected orexinergic and glutamatergic neurons with synaptic diversity. Diversity, as opposed to noise, improved stability of the sleep-wake cycle.	
Williams & Diniz Behn 2011 [159]	Investigated effects of local neuromodulator, dynorphin, on spiking activity of sleep- and wake-stabilizing orexin neurons. Predicted that the delay in functional effects of orexin neurons at the transition from sleep to wake is due to desensitization of orexin neurons to dynorphin.	Hodgkin-Huxley type neurons; 2 neurons with transmitter-gated synapses
Carter et al., 2012 [157]	Simulated detailed hypocretin neuron in lateral hypothalamus and noradrenergic neuron in locus coeruleus (LC) coupled via chemical synapses. Demonstrated that release of hypocretin increased excitability of LC neurons and that LC neurons are critical for integration of hypocretin effects on wake-NREMS transitions.	Hodgkin-Huxley type neurons; 2 neurons [157], 40 neurons [158]; 60 neurons [109]
De Lecea & Huerta 2014 [158]	Icreased the number of both hypocretin and LC neurons and simulated optogenetic excitation of hypocretin neurons leading to slow depolarization and spiking of LC neurons.	
Mosqueiro et al., 2014 [109]	Investigated the interplay between slow action of hypocretin and fast action of GABAa in the hypocretin-LC control of sleep-wake cycles. Predicted the need for slow inhibitory neuropeptides acting on time scales similar to hypocretin.	

References are from the list in the main document.
